# Young adults' return migration from large cities in Sweden: The role of siblings and parents

**DOI:** 10.1002/psp.2354

**Published:** 2020-07-02

**Authors:** Clara H. Mulder, Emma Lundholm, Gunnar Malmberg

**Affiliations:** ^1^ Population Research Centre, Faculty of Spatial Sciences University of Groningen Groningen Netherlands; ^2^ Centre for Demographic and Aging Research and Department of Geography Umeå University Umeå Sweden

**Keywords:** parents, return migration, siblings, Sweden, young adults

## Abstract

Living in cities affects young adults' access to education and work. With the use of register data for 2000–2013, we examined the role of having siblings and parents living close by and having siblings and parents living in the area of origin, in young adults' return migration from the four largest cities in Sweden. We found that young adults were less likely to return, and also less likely to migrate elsewhere, if they had siblings or parents living in the city of residence than if this was not the case. If the parents no longer lived in the region of origin, the young adults were very unlikely to return. Young adults were more likely to return if they had siblings living in that region than if they had no siblings or the siblings lived elsewhere. Adverse circumstances such as dropping out of tertiary education, low income, and unemployment were associated with a greater likelihood of return migration.

## 1 INTRODUCTION

Migration to a large city is a major step in the life courses of many young adults in developed countries. Making this step, and subsequently spending an episode in a large city, allows them to make use of the ample opportunities for education and work that such cities tend to offer. For some of these young adults, the city will turn out to be attractive enough to remain there for a long time; others will move back to their home region or move on to other locations. (Note: We use the terms “home region” and “region of origin” interchangeably to refer to the previous region of residence of the young adult, where at least one parent also lived before the young adult moved.) Clearly, the outcomes of the location choices of young adults who moved to cities—staying in the city, moving back, and moving on—are important in many ways. For the young adults themselves, these location choices greatly affect their access to education, work, housing, amenities, and social networks. For example, among young Americans, returning to the parental home was associated with negative economic outcomes (Sironi & Billari, 2019). (Note: Our interest is in return migration to a previous region, not necessarily to the parental home. However, because returning to the home region coincides with returning to the parental home for quite a few young adults, we also refer to some of the literature on returning to the parental home.) For cities, home regions, and other destination regions, these choices affect the availability of human capital, the size and structure of the population, and the attendant demand for housing, education, and jobs. Indeed, as Von Reichert, Cromartie, and Arthun (2011) argue, “return migrants can be a boost to the economic and social vitality of rural communities and that communities should make efforts to both attract and retain them” (p. 35).

Given this importance of young adults' location choices, our interest is young adults' migration from large cities after having moved there previously—either to return to their region of origin or elsewhere. In line with other work emphasising the role of family in internal migration (summarised by Mulder, 2018; see also Thomas, Gillespie, & Lomax, 2019 on family motives for migration), several recent studies have looked into the role of family in return migration. These studies have established that family reasons form a substantial share of the motivations of young return migrants (Haartsen & Thissen, 2014) and that the location of parents is crucial in young adults' return migration (Zorlu & Kooiman, 2019; see also qualitative work by Von Reichert, Cromartie, & Arthun, 2013). We contribute to this line of research by investigating the importance of siblings in return and onward migration, while also taking into account the role of parents. We address the following research question: *To what extent is return migration from large cities in Sweden (as opposed to staying in the city or migrating onward) associated with the presence of siblings and parents in the city and in the region of origin?*

We use Swedish register data for the entire Swedish‐born population of young adults who moved to Stockholm, Gothenburg, Malmö/Lund, or Uppsala in 2000–2012 between the ages of 18 and 28 and follow them until migration, age 36 or censoring in 2013. We analyse these data using multinomial logistic regression of returning to the local labour‐market area of origin or migrating elsewhere versus not migrating. A fourth category in the model consists of those moving from the city at a shorter distance than 50km. Because such moves are not of substantive interest to our research question, we do not present results for this category.

## 2 THEORETICAL AND RESEARCH BACKGROUND

When theorising about the role of parents and siblings in return migration, it is important to distinguish between parents and siblings living in the city of residence and those living in the region of origin. For parents and siblings living in the city of residence, there is no a priori reason for expecting a different role in return migration than in migration to other areas. We therefore discuss their role in remaining in the city in a separate section. We then turn to return migration as a specific type of migration before we go into the role of parents and siblings living in the region of origin in return migration.

It should be borne in mind that the population we study are those young adults who moved to one of the four cities from a region in which one of their parents lived before the young adults moved. This implies that there are two ways in which parents may have ended up in the young adult's city of residence: One parent may have already lived in the city before the young adult moved, or one or both parents may have moved to the city during the period of observation.

### 2.1 The role of parents and siblings living in the city of residence in remaining there

As has been shown in, for example, the literature on family solidarity (Bengtson, 2001), family members are known to be important social network members (see also Rossi & Rossi, 1990). Although usually not as close as the relationships between parents and children (Bengtson, 2001), relationships among siblings tend to be close as well (Cicirelli, 1995; Voorpostel & Blieszner, 2008) and frequently involve support exchange (Eriksen & Gerstel, 2002; Voorpostel & Van der Lippe, 2007; Weaver, Coleman, & Ganong, 2003). Therefore, parents and siblings living in the city may encourage young adults to remain in the city and thus lead to a lower propensity to migrate. As social network members, both parents and siblings may provide insider information about the city, as well as support and companionship. They may thus facilitate social integration in the city and help the young adult overcome feelings of loneliness. Because of the closer relationships between parents and children than among siblings, and because parents usually have more resources than siblings, it is likely that parents are more important to staying in the city than siblings. Yet, as age peers, siblings perform specific functions, such as offering services, teaching each other skills and abilities, and regulating each other's behaviour (Weaver et al., 2003).

A few previous studies have shown that having parents living close by is associated with a decreased likelihood of migration (e.g., Ermisch & Mulder, 2019, for the United Kingdom; Michielin, Mulder and Zorlu 2008 and Zorlu, 2009, for the Netherlands; Mulder & Malmberg, 2011, 2014, for Sweden). Of these studies, those by Zorlu and by Mulder and Malmberg also took into account whether siblings lived close by and demonstrated that the location of siblings mattered in a similar way as that of parents.

In accordance with these theoretical considerations and research findings, our first hypothesis reads: *Young adults will be less likely to return from a city to their region of origin*—*and also less likely to migrate onward*—*if a parent or sibling lives in the city (H1).*

### 2.2 Return migration as a specific type of migration

From the 1980s onward, scholars have acknowledged that return migration differs from other types of migration in several ways (e.g., DaVanzo & Morrison, 1981). As noted by Haartsen and Thissen (2014), much of the literature on return migration relies on the success–failure dichotomy: Some return moves can be seen as a sign of success, for example, if people “step off the escalator” when returning from an urban area in which they experienced upward mobility (compare Fielding, 1992; Champion, 2012), whereas other return moves are made to correct a previously unsuccessful move (Hunt, 2004). The success–failure dichotomy is useful because it emphasises that return migration may be related to socio‐economic resources in a different way than migration in general and onward migration. Whereas migration in general tends to be associated with higher levels of education and enrolment in education, return migration may just as likely be triggered by adverse circumstances such as dropping out of education, low income, or unemployment. Although our main hypotheses are related to the role of parents and siblings in return migration, we therefore also pay attention to the possible roles of adverse circumstances (in terms of the success–failure dichotomy, these could be seen as “failures”) and positive incentives for return and onward migration in terms of graduation from post‐secondary education and high levels of education (which in terms of the success–failure dichotomy could be seen as signs of success).

Despite its usefulness, the success–failure dichotomy has been criticised for being incomplete and being focused too much on economic reasons for migrating (e.g., Haartsen & Thissen, 2014). For example, Haartsen and Thissen (2014) note that return migration may be planned ahead. For example, young adults may leave their region to get an education and return as soon as they graduate. Importantly, as Niedomysl and Amcoff (2011) concluded from analyses of a Swedish survey, return migration stands out from other types of migration in that it is largely driven by social considerations rather than work or education. Based on interviews with return migrants to rural communities in the United States, Von Reichert, Cromartie, and Arthun (2014; see also their 2013 article) concluded that three considerations played a part in return migration: employment (which included returning to a family farm or business), family living in the region of origin, and ties to the community.

### 2.3 The role of parents and siblings living in the home region in return migration

Just like parents and siblings living in the city will likely encourage remaining there, parents and siblings living in the home region will likely be attraction factors for return migration. Undoubtedly, an important part of the return migration of young adults can be regarded as “returning home” to parents, either to live with them or to live close to them; to find support or company, and sometimes possibly to provide support to them. (Note: Because our study population is rather young and previous research has shown that those under 40 are much more likely to move towards parents for their own needs than for their parents' needs [e.g., Smits, 2010; Smits, Van Gaalen, & Mulder, 2010], we refrain from investigating the associations between return migration and parental support needs.) Co‐residence in the parental home has been identified as an important form of support from parents to young adult children, albeit more so in Southern European than Nordic countries (Albertini & Kohli, 2013). Previous studies from Britain indicated that returning to the parental home (“boomeranging”) was related to economic dependency and turning points in the life course, for example, partnership dissolution (Stone, Berrington, & Falkingham, 2014). Similar results were reported in a study on returning to the parental home in Sweden, while returning to parental neighbourhoods (not moving in with the parents) was associated with more independent economic positions (Olofsson et al., published online before print).

The importance of parents to return migration to a previous region was indeed highlighted in Zorlu and Kooiman's (2019) study on return migration in the Netherlands. The likelihood of returning to the “home region” was found to be much higher for those whose parents still lived in the region than for those whose parents had moved out or were no longer alive. According to Von Reichert et al. (2013), family relationships played an important part in their study participants' motivations for return migration. The return migrants first and foremost mentioned their parents as the focal point of the return move. Conversely, the authors concluded from interviews with participants who had *not* returned that “if the parents had moved away, the incentive and inclination to return was greatly diminished and practically eliminated, as out‐migrants repeatedly stated: There is nothing here. My parents don't live here, and there are no jobs” (Von Reichert et al., 2013, p. 262).

Next to parents, siblings living in the parental home or the region of origin could contribute to the feeling of “home” associated with this region. Indeed, participants in the fieldwork by Von Reichert et al. (2013, 2014) not only mentioned parents but also frequently brought siblings to the fore as a consideration in return migration. In a study based on Swedish register data not specifically focusing on return migration, Pettersson and Malmberg (2009) found that siblings formed an extra attraction factor for moving towards older parents. Furthermore, Mulder, Lundholm, and Malmberg (accepted for publication) found that young adults were more likely to move to large cities in Sweden if they had a sibling living there. In accordance with these considerations and findings, our second hypothesis reads: *Young adults will be more likely to return from a city to their region of origin if a parent or sibling lives in that region (H2).*

### 2.4 Adverse circumstances and indicators of success

Return migration, and particularly moving back to live with family or close to family, could be related to a need for assistance or comfort in adverse circumstances. Indeed, previous research has shown that moving close to parents (Smits, 2010), moving in with parents (Smits et al., 2010), and returning home to live with parents (Stone et al., 2014) were associated with adult children's support needs. Given the young age of the study population and the available information in our data, we focus on dropping out of education, income, and unemployment and hypothesise that *young adults who drop out of education, those who have lower incomes, and those who are unemployed will be more likely to return from a city to their region of origin than others (H3).* We also investigated whether the role of the residential locations of siblings and parents was greater for these young adults.

Just like adverse circumstances, successful completion of education could also lead to return migration, for example, if the return was already planned before the move to the city (Haartsen & Thissen, 2014) or if the young adults find employment in the home region after obtaining a high level of education (compare Stone et al., 2014). This idea leads us to hypothesise that *young adults who graduate from post‐secondary education, and others with a degree, will be more likely to return from a city to their region of origin than others (H4).*

### 2.5 Other factors associated with return and onward migration

Naturally, we need to account for other factors associated with migration—either return migration or migration in general. One could expect young adults to be more likely to move from the smaller cities Gothenburg, Malmö/Lund, and Uppsala (with fewer educational and labour‐market opportunities) than from Stockholm. Women may be less likely to return but more likely to migrate elsewhere (compare Smits, 2010; Smits et al., 2010). Migration is known to be highly age structured (Bernard, Bell, & Charles‐Edwards, 2016). Highly educated young adults are more likely to migrate than those with less education (Lundholm, 2007), but this may be reversed for return migration (Zorlu & Kooiman, 2019). We also account for income and household status (Zorlu & Kooiman, 2019). We may expect those who have a history of living in or near the city of residence before they moved there—or whose parents have such a history—to be less likely to return to their previous region of residence and also to move elsewhere. A history of living elsewhere than the city of residence and the previous region of the young adult or the parents could lead to a smaller likelihood of returning but a greater likelihood of migrating elsewhere (see Bernard & Vidal, 2020, on the impact of migration histories). With regard to parental characteristics, we also account for their level of education and income: Parents with more resources could encourage their children to stay in the city or move on but could also be more attractive to return to (see Avery, Goldscheider, & Speare, 1992, for the “feathered‐nest” hypothesis). One could expect young adults to be more likely to return to more attractive regions offering better opportunities for work and education. To capture the attractiveness of the region of origin, we account for the distance to the place of residence in that region before the young adult moved to the city of residence, that region's population size, whether it has opportunities for higher education, and the unemployment rate. Finally, we account for changes in levels of migration over time connected with, for example, business cycles.

## 3 DATA AND METHODS^1^

### 3.1 Dataset

We used Swedish register microdata covering all residents registered in the country, provided by Statistics Sweden. The data included the area of birth (county level) and annually updated socio‐economic information and residential locations. The data also contained links to parents and to siblings (including half‐siblings but not step‐siblings) via the parents.

Our study population consisted of those young adults aged 18–28 who moved to one of Sweden's four largest cities in the period 2000–2012^2^: Stockholm, Gothenburg, Malmö/Lund, and Uppsala. The age range of 18–28 was chosen because in Sweden, this is the life‐course span in which migration propensities are highest (Lundholm, 2007), and most of the increases in distances between parents and siblings take place (Kolk, 2017). Two further requirements were that they had to originate from areas outside the Local Labor Market areas (LLMs as defined by Statistics Sweden) containing these cities and that at least one of their parents lived in the same region. Both those living in the parental home and those living in the same LLM as their parents but away from the parental home were included in the analysis. The city of Stockholm was defined as the municipalities that are fully or partly included in the urban area of Stockholm, that is, Solna, Sundbyberg, Sollentuna, Täby, Nacka, Huddinge, Botkyrka, Järfälla, Haninge, Danderyd, Tyresö, and Stockholm. For Gothenburg, we used the municipalities of Gothenburg and Mölndal; for Malmö/Lund, the municipalities of Malmö and Lund; and for Uppsala, the municipality of Uppsala. We grouped the cities of Malmö and Lund because of the short distance between these cities (around 20km).

We followed the young adults until they left the city (either to return to the LLM of origin or to move elsewhere), the end of the observation period (2013) or when they reached the age of 36. This age limit was chosen to ensure some homogeneity in life‐course phase and a sufficient number of observations at each age. We excluded immigrants (around 9%) because fewer than half of them could be linked to a mother in Sweden, and level of education was frequently missing for them. We also excluded less than 1% of the cases for which level of education was missing for the index person or for both parents. In total, 112,097 person‐years were included in the analysis in which 6,979 return moves, 4,587 moves elsewhere of 50+km, and 1,758 moves elsewhere of less than 50km were observed.

We analysed moves between end of December of 12 pairs of years t0 and t1: 2001–2002 up to 2012–2013.

### 3.2 Variables

Our migration variable consisted of four categories: returned to the LLM of origin between t0 and t1, migrated elsewhere over distances of 50km or more, and moved elsewhere over distances shorter than 50km, as opposed to stayed in the current region of residence (reference category). The category “moved elsewhere over distances shorter than 50 km” was a residual category.^3^ For descriptive purposes, we also distinguished those who returned to the parental home from those who returned to the previous LLM but not the parental home, using an indicator of household type provided by Statistics Sweden. However, this distinction was not possible for those with children: According to the definition used for this indicator, a household can only consist of two generations, which implies that as soon as a person becomes a parent, the new parent–child dyad will be recorded as a separate household next to the household of the parent(s).

With a few exceptions (see below), all independent variables were measured at t0. Our main independent variables were indicators of whether a sibling, a parent, or both a sibling and a parent lived in the city of residence versus none (reference category) and the same indicators for the home region. Naturally, there was some collinearity between these two indicators. We therefore also tried other specifications of the main independent variables, such as indicators measuring whether parents and siblings lived in the home region, in the city of residence, in both, or in neither. The results were robust to the different specifications.

In some cases, one parent may already have lived in the city at the time the young adult moved there. This parent may have formed an attraction factor for moving to the city in the first place. In other cases, the young adult may have moved to the city together with the parents or at the same time, or one or both parents may have moved to the city after the young adult moved there. For additional analyses, we also used indicators of whether a sibling was living in the city who was a sister, of similar age (between 3 years younger and 3 years older), more than 3 years older, a sibling who had completed post‐secondary education, and a sibling who was enrolled in education.

At the level of the individual, we further included a categorical variable for whether the index person lived in the city of Gothenburg, Malmö/Lund, Uppsala, or Stockholm (reference), an indicator for whether the index person is a woman and a categorical age variable (18–21, 22–24, 25–29, 30–35). Another categorical variable was a combined measure of level of education and whether the index person was a student. For students at t0, we used information from both t0 and t1. The categories were no university degree, not a student at t0 (reference category); student at both t0 and t1 (continuous student); student at t0, no longer student at t1, and no university diploma at t1 (student who dropped out of university); student at t0 but not at t1, having a university diploma at t1 (student who graduated); student at t0 and t1 having a university degree at t1 (student studying beyond first degree, at advanced level); and university degree at t0, not a student at t0 or t1. In this way, we could identify students and transitions out of education with or without a degree. Individual disposable income (in 100,000s of Swedish crowns) was derived from the tax register; the few registered negative incomes were recoded to 0. Enrolment in education and unemployment was derived from information about annual income from student allowances (including student loans) and unemployment benefits. If the index person received any income from these sources during a year, they were coded as student or unemployed. This implies that the dummy variable for unemployment should be interpreted as an indicator of having an insecure labour‐market position.

We used a categorical variable to measure whether the index person was unmarried without children (reference), was married without children, lived with a partner (either married or unmarried) and children, or lived with children but not with a partner. Unfortunately, the data did not allow us to identify unmarried cohabitation (which is in fact very common in Sweden) for those without children. The previous migration history was derived from the county of birth and was coded as born in the county containing the LLM of origin, born in the county containing the city of residence, or born elsewhere in Sweden. Those born outside Sweden were not included.

At the level of the family of origin, the parents' migration history measured whether at least one parent was born in the county of the city of residence or, if this was not the case, elsewhere in Sweden or abroad (reference: both parents born in the county containing the LLM of origin). The parents' completed level of education was measured in three categories: primary, secondary, and post‐secondary education. Parental income was based on the same measure as for the index person. For education, we used the highest known level of the two parents, and for income, we added the two incomes.

The distance between the city of residence and the original place of residence was measured in 100km. At the level of the LLM of origin (Sweden has 70 LLMs), we further used indicators of population size in 100,000s at t0, whether there was a full university or, if not, a university college (that is, a higher education institute that offers only a limited number of disciplines and does not hand out PhD degrees) in the LLM and the unemployment rate. Finally, we included a categorical variable for year of observation: 2001–2008 (reference), 2009–2010, and 2011–2012. These three periods capture economic cycles, with a stable period followed by a period characterised by an economic downturn and finally a period of recovery after the crisis. Descriptive statistics for all variables and migration percentages per person‐year across the categories of the categorical independent variables are presented in Table 1.

**TABLE 1:** Descriptive statistics and percentages migrating (*N* person years = 112,097; means in italics)

	% in sample/*mean*	SD	Not moving	Return move	Migration elsewhere	Move elsewhere <50km
Migrated (dependent variable)			88.11	6.23	4.09	1.57
Parents or siblings in destination city: neither	72.31		87.16	7.00	4.25	1.59
Parents only	2.58		90.17	3.81	4.36	1.66
Siblings only	21.20		90.27	4.60	3.58	1.54
Both parents and siblings	3.92		92.76	2.32	3.76	1.16
Parents or siblings in home region: neither	5.68		92.09	1.05	5.23	1.62
Parents only	33.37		89.61	4.79	4.02	1.58
Siblings only	1.17		89.25	1.83	6.71	2.21
Both parents and siblings	59.78		86.88	7.60	3.97	1.54
City of residence: Stockholm	41.60		90.30	5.39	2.87	1.45
Gothenburg	31.82		87.61	6.49	3.97	1.93
Malmö/Lund	15.13		86.29	7.11	4.91	1.69
Uppsala	11.45		84.00	7.37	7.79	0.84
Woman	55.17		87.36	6.62	4.33	1.68
Age category: 18–21	18.80		82.03	12.14	4.80	1.03
22–24	22.74		85.53	8.06	5.29	1.12
25–29	35.62		89.62	4.55	4.13	1.70
30–35	22.85		93.35	2.15	2.26	2.24
Education/student status: low/not student	35.33		87.09	7.23	3.65	2.04
Student (continuous)	14.03		86.42	8.28	4.62	0.68
Student (dropping out)	2.76		80.49	14.51	3.98	1.03
Student (graduating)	7.00		89.33	7.10	3.04	0.53
Advanced student	14.86		85.63	7.86	5.90	0.62
Highly educated, not student	38.17		90.98	3.16	3.80	2.06
Income (SEK 100,000 s)	*1.70*	*1.35*	*1.74*	*1.23*	*1.40*	*1.98*
Unemployed	8.22		86.73	7.27	4.43	1.57
Household status: unmarried, no children	83.19		87.50	6.79	4.45	1.26
Married, no children	3.79		90.10	3.48	3.29	3.13
Partner^a^ and child(ren)	12.03		91.45	3.34	2.01	3.21
Child(ren), no partner	0.98		91.88	4.20	2.19	1.73
Migration history: born in county of origin	74.66		87.76	6.63	4.02	1.59
Born in city of residence	9.64		90.48	4.49	3.30	1.73
Born elsewhere	15.70		88.35	5.37	4.93	1.35
Parents' migration history: no migration history	33.57		87.36	7.05	3.98	1.61
Either born in destination county	12.25		89.46	5.21	3.55	1.78
Born elsewhere, neither in destination county	42.91		87.99	6.02	4.44	1.54
Born abroad, neither in destination county	11.27		89.36	5.64	3.68	1.32
Parents' highest level of education: primary	5.71		88.65	6.29	3.19	1.87
Secondary	37.52		87.94	6.51	3.71	1.84
Tertiary	56.76		88.17	6.03	4.43	1.36
Parents' income (SEK 100,000s)	*4.98*	*11.50*	*5.00*	*4.63*	*5.19*	*4.65*
Distance to original place of residence	*2.77*	*1.99*	*2.75*	*2.86*	*2.93*	*2.80*
Population size in home region (100,000s)	*1.38*	*0.68*	*1.39*	*1.39*	*1.34*	*1.34*
University in home region: no	26.95		87.96	5.88	4.52	1.65
Full university	24.54		88.77	5.91	3.74	1.57
University college	48.51		87.87	6.58	4.03	1.52
Unemployment rate in home region	*17.36*	*6.21*	*17.18*	*19.41*	*18.37*	*16.86*
Year: 2001–2008	79.77		86.88	7.27	4.39	1.46
2009–2010	11.79		92.30	2.44	3.35	1.91
2011–2012	8.44		93.88	1.64	2.34	2.15

^a^ Unmarried partners can only be identified for those with children.

### 3.3 Analytical strategy

We employed multinomial logistic regression models of returning from the city of residence to the LLM of origin, migrating 50+km to elsewhere in Sweden versus staying in the city (reference). In the models, there was also a fourth category for moving elsewhere over a distance smaller than 50km, but because this category is not of interest to our research question, we do not show the results. The standard errors were corrected for the clustering of index persons in LLMs (the highest‐level unit of analysis at which variables were measured).

Next to the models we present, we also ran several additional analyses, for example, including interaction terms. We discuss the results of these analyses briefly without showing them in tables. We also discuss the results of some additional descriptive analyses in which we explored different specifications of the main independent variables.

## 4 RESULTS

### 4.1 Descriptive findings

Overall, a return move to the home region took place in 6.2% of the observed person‐years and an onward move in 4.1% (Table 1). In terms of persons, 39.0% returned, and 25.7% migrated onward; another 9.8% moved from the city of residence over a shorter distance (not in table; this also holds for percentages of returns mentioned below in this section). From our—imperfect—indicator of returning to the parental home rather than just the region, we estimated that 55% of the returns to the region (or 3.4% of all person‐years) were also returns to the parental home. The percentage returning to the parental home is higher than Olofsson et al. (published online before print) found, also for Sweden. Olofsson et al.'s estimate was 2.6% of person‐years. This difference may partly be due to a difference in observation period (theirs was 1986–2009) but likely also to the fact that we focus on a specific category of young adults—those who moved to one of the large cities—whereas they observed complete birth cohorts.

Returns to the parental home were overrepresented among those younger than 25 before the potential move (67% of the returns) and students: 74% among continuous students, 64% among students who dropped out of university, and 70% among advanced students. They were underrepresented among those who already had a university degree at t0 (30% of the returns). It is likely that many of those who returned to the parental home left home again after a short period of time. Some of them may not even have returned but may have registered with their parents while actually living at some temporary address or abroad.

The percentage returning was considerably smaller if parents or siblings lived in the city: 3.8% of person‐years if at least one parent lived in the city of residence but not a sibling, 4.6% if at least one sibling lived in the city but not a parent, and 2.3% if both a parent and a sibling lived there (Table 1). For migrating elsewhere, the differences were smaller. The differences in the percentage returning according to whether parents or siblings lived in the region of origin were even larger. In the rare situation (5.7% of the person‐years) that neither a parent nor a sibling lived in the region of origin, a return was observed in only 1.1% of the person‐years. In the even rarer situation that only siblings lived there (1.2% of the person‐years), the percentage returning was 1.8 of person‐years, compared with 4.8% returning if only at least one parent lived in the home region (which was the case in 33.4% of the observed person‐years) and 7.6% if both a parent and a sibling lived there (59.8% of the observed person‐years). Thus, for the vast majority of young adults, returning to the home region coincides with moving close to parents, and for many, it also coincides with moving close to siblings. This finding resonates well with Von Reichert et al.'s (2013) quote above about the practical elimination of the incentive to return if the parents no longer lived in the home region. It should be borne in mind that our study population comprises only those young adults who lived in the same region as at least one parent before the move. Therefore, in those cases in which neither parent lived in the home region, the parent or parents moved out of that region after the young adult did.

To investigate in more detail how the percentage returning differed according to the residential locations of parents, we also explored different specifications of the indicators of where the parents lived (percentages not shown in tables). These explorations revealed that somewhat higher percentages returned when only the mother lived in the region of origin (5.9%) than only the father (4.7%), and the highest percentage returned if both parents lived there (6.9%). If both parents lived in the home region, it did not make a difference whether both parents lived together (6.8% returned) or had separated (6.7% returned).

A particularly high percentage returning (14.5%) was found among those young adults who left education without a diploma (Table 1). By contrast, no great difference in the percentage returning was found between the unemployed (7.3%) and others (6.1%). Another noteworthy descriptive finding was that a smaller proportion of young adults moved from Stockholm than from the other cities. There were fewer moves in the later periods than in 2001–2008, but this is mostly related to the study design: In these later periods, a greater proportion of index persons had stayed in the city for a longer time and a greater proportion were older.

### 4.2 Model findings: The role of parents and siblings in return and onward migration

The model findings presented in Table 2 confirm the importance of the locations of parents and siblings in return migration. As expected, young adults who had at least one parent or sibling living in the city of residence were less likely to return to the LLM of origin than those who did not. For having only at least one parent in the city the parameter was −0.36 (implying an odds ratio of exp[−0.36] = 0.70; *p* = .00). For having only one or more siblings, the parameter was −0.26, with *p* = .00. The difference between these two parameters is not very large and not statistically significant (*p* = .28). The parameter for having both at least one parent and at least one sibling in the city of residence was −0.63, which is about twice as large as each of the other parameters. These findings suggest a cumulative effect of having a parent and a sibling in the city of residence on return migration. For onward migration, the parameter for having only a parent in the city of residence was close to zero (−0.04, with *p* = .73), but statistically significant associations were found with having only a sibling in the city of residence (−0.14, *p* = .00) and having both parents and siblings living there (−0.22, *p* = .03). To conclude, except for onward migration if a parent lived in the city of residence, we found support for Hypothesis 1 that young adults are less likely to return and to a lesser extent also to migrate onward if parents and/or siblings live in the city of residence.

**TABLE 2:** Multinomial logistic regression of returning from the city or migrating elsewhere, versus staying in the city

	Returned			Migrated elsewhere		
	coeff	SE	*p*	coeff	SE	*p*
Parents or siblings in destination city (ref. neither)						
Parents only	−0.358***	0.086	.000	−0.037	0.106	.726
Siblings only^a^	−0.257***	0.038	.000	−0.143***	0.035	.000
Both parents and siblings^b^	−0.632***	0.111	.000	−0.224^*^	0.100	.025
Parents or siblings in home region (ref. neither)						
Parents only	1.344***	0.129	.000	−0.306***	0.060	.000
Siblings only^c^	0.469^*^	0.218	.032	0.298^*^	0.140	.033
Both parents and siblings^d^	1.586***	0.132	.000	−0.362***	0.060	.000
City of residence (ref. Stockholm)						
Gothenburg	0.067^*^	0.032	.036	0.271***	0.056	.000
Malmö/Lund	0.123^**^	0.043	.004	0.415***	0.066	.000
Uppsala	0.107^*^	0.044	.015	0.874***	0.048	.000
Woman	−0.009	0.028	.754	0.083^**^	0.026	.002
Age category (ref. 18–21)						
22–24	−0.372***	0.029	.000	0.020	0.051	.695
25–29	−0.803***	0.035	.000	−0.174^**^	0.068	.010
30–35	−1.227***	0.069	.000	−0.485***	0.087	.000
Education/student status (ref. low/not student)						
Student (continuous)	−0.595***	0.049	.000	−0.312***	0.059	.000
Student (dropping out)	0.357***	0.060	.000	−0.199^†^	0.103	.054
Student (graduating)	−0.616***	0.052	.000	−0.762***	0.077	.000
Advanced student	−0.149^*^	0.060	.013	−0.027	0.048	.573
Highly educated, not student	−0.255***	0.040	.000	0.175***	0.048	.000
Income (SEK 100.000 s)	−0.401***	0.031	.000	−0.260***	0.023	.000
Unemployed	0.135^**^	0.047	.004	0.068	0.061	.269
Household status (ref. single)						
Partner, no children	−0.059	0.087	.496	−0.160^†^	0.092	.084
Partner and child(ren)	0.211^**^	0.073	.004	−0.429***	0.080	.000
Child(ren), no partner	0.122	0.119	.305	−0.439^*^	0.218	.044
Migration history (ref. born in origin)						
Born in city of residence	−0.178^**^	0.058	.002	−0.114^†^	0.066	.083
Born elsewhere	−0.062^†^	0.035	.074	0.122^**^	0.042	.004
Parents' migration history (ref. no migration history)					
Either born in destination county	−0.161***	0.051	.001	−0.075	0.069	.280
Born elsewhere, neither in destination county	−0.070^*^	0.032	.028	0.031	0.037	.395
Born abroad, neither in destination county	−0.222***	0.040	.000	−0.149^*^	0.063	.017
Parents' highest level of education (ref. low)						
Middle	−0.077	0.056	.168	0.084	0.073	.249
High	−0.095^†^	0.053	.075	0.155^**^	0.060	.010
Parents' income (SEK 100.000 s)	−0.003	0.002	.188	0.001***	0.000	.000
Distance to original place of residence	0.006	0.008	.480	0.043***	0.010	.000
Population size in home region (100.000 s)	0.082^†^	0.048	.091	0.033	0.043	.440
University in home region (ref. no)						
Full university	0.041	0.098	.674	−0.197^**^	0.072	.007
University college	0.089	0.068	.188	−0.100	0.063	.112
Unemployment rate in home region	0.021***	0.004	.000	0.005^†^	0.003	.093
Year (ref. 2001–2008)						
2009–2010	−0.400***	0.065	.000	−0.072	0.056	.204
2011–2012	−0.453***	0.097	.000	−0.291***	0.070	.000
Constant	−1.646***	0.141	.000	−2.792***	0.168	.000
N	112,097					
Log pseudolikelihood	−50679.1					

*Note*: Results for short‐distance moves elsewhere not shown. *p* values for difference with “parents only” (return, elsewhere):

^a^ .000, .000;

^b^ .000, .025;

^c^ .032, .033;

^d^ .000, .000.

†; *; **; ***

In additional analyses, we explored whether it mattered if a sibling living in the city of residence was a sister or a brother, older, of similar age or younger, highly educated or a student (results not shown), but we did not find indications that that was the case.

Very pronounced associations were found between having a parent or sibling living in the region of origin and the likelihood of return migration. Compared with those having neither a sibling nor a parent in the home region, those who had a parent living there were exp(1.34) or 3.83 times as likely to return, those who had a sibling there exp(0.47) or 1.60 times, and those who had both a parent and a sibling living there exp(1.59) or 4.89 times. The differences between the parameters for siblings only and parents only, and between those for both siblings and parents and parents only, were substantial and statistically significant. These findings suggest that parents are a more important factor in return migration than siblings and that siblings form an additional attraction factor next to parents (compare Pettersson & Malmberg, 2009). Thus, Hypothesis 2 is supported. Onward migration was less likely if a parent lived in the home region (also in combination with siblings) than if no parent lived in that region, possibly because parents elsewhere may attract migration. According to the model, those who only had siblings living in the home region were more likely to migrate onward than those who had neither parents nor siblings living there. However, because only having siblings in the home region was a very rare situation (see Table 1), we refrain from trying to interpret this finding.

### 4.3 Model findings: Adverse circumstances and successful graduation

Young adults who left education without a diploma were considerably more likely to return to the home region than those in any other category of our variable indicating level of education, student status, and changes therein. The negative parameter for income indicates a greater likelihood of returning for those with lower incomes. All else equal, unemployed young adults were also more likely to return than others. Thus, in line with Hypothesis 3, adverse circumstances were associated with a greater likelihood of return migration. This is also true in comparison with onward migration.

We also investigated whether parents and siblings had a greater role in the likelihood of returning for those in adverse circumstances related to education, income, and unemployment. We ran several models including interaction terms between our indicators of the residential locations of parents and siblings and our indicators of adverse circumstances, but none of the interaction parameters were substantial or had *p* values below .05. Furthermore, the directions of the estimations were sensitive to the exact specification of the independent variables. This might indeed imply that adverse circumstances tend to lead young adults to return to previous places of residence irrespective of whether there are parents or siblings to return to at these previous locations. Yet, we should be cautious interpreting this lack of interaction effects. As we saw earlier, the vast majority of young adults had parents and/or siblings living in the region of origin. For those who returned, this majority was even more overwhelming; this is also true of those in adverse circumstances who return.

In contrast with adverse circumstances, we do not find evidence that graduation and a high level of education are associated with a higher likelihood of return migration. Thus, we find no support for Hypothesis 4. Those who graduated in the year of observation were less likely to return than any other category in the variable indicating level of education and student status. It is noteworthy that, except for dropping out of university, all other categories (continuing students, advanced students, and those who had finished tertiary education) were less likely to return than those who were less educated and not enrolled in education. The pattern for onward migration was different: Those with a university degree were most likely to make such a move.

### 4.4 Model findings: Other

The model findings confirm that both return and onward migration occurred more frequently from the other cities than from Stockholm. No gender difference was found in the likelihood of return migration, but women were more likely to migrate onward than men. It could be that some of these women migrated towards partners. Both return and onward migration were less common at older ages. The findings for marital status and the presence of children in the household also differed between return and onward migration: Those who were married or had children were less likely to migrate onward than others, but return migration was most likely among those with a partner and children. These differences in findings between return and onward migration further illustrate the different characters of these two types of migration.

As one would expect, those who were born in the city of residence, or whose parents were born there, were less likely to return to the LLM of origin. This was also the case for those who were born (or whose parents were born) in a different LLM and for those whose parents were born abroad. Parental level of education had few pronounced effects, although those whose parents had completed tertiary education were somewhat more likely to migrate onward. Neither were there strong associations with characteristics of the home region, with the surprising exception of the unemployment rate in the home region: This rate was positively associated with the likelihood of returning. Finally, among this study population of young adults who lived in cities after moving there from other regions, the annual likelihood of return migration, and to a lesser extent also onward migration, decreased over time.

## 5 CONCLUSIONS AND DISCUSSION

A small but growing literature shows that non‐resident family is an important factor in internal migration. To this literature, we contribute an investigation of the importance of siblings in young adults' return migration from large cities to the region in which they, and at least one of the parents, lived before they moved to the city. We also took into account the role of parents. As in much of the previous work on return migration, we also looked at onward migration.

Our findings confirm the importance of the locations of siblings and parents in young adults' migration behaviour. Siblings and parents living in the city were associated with substantial decreases in the likelihood of return migration, with the results suggesting similar importance of siblings and parents. For the home region, the findings were different: Having at least one sibling living there played a part in the likelihood of returning, but the part of having at least one parent living there was paramount. Without much exaggeration, we could assert that, for young adults who move to large cities in Sweden from a region where at least one parent also lives, return migration almost always equals returning to parents—either to live with them or to live close to them. This can be derived from the very strong positive effect of having parents living in the home region on the likelihood of returning there, in combination with the finding that only a small proportion of parents moved from the home region after the young adult did. This finding suggests that, for this population of young adults, parents are a major attraction factor for return migration. At least among our study population, it seems that attachment to the home region and location‐specific capital left behind there are rarely a sufficient reason for returning.

We also found a greater likelihood of return migration among those in adverse circumstances—dropping out of university, low income, and unemployment. We did not find evidence of a greater role of the residential location of siblings or parents in return migration in such circumstances. This could be because of the very important role of parents and siblings in the first place—not only among those in adverse circumstances but also among all young adults. We did not find any evidence of a greater likelihood of returning after graduation, among those with higher education, or among those with higher income—rather, these factors are associated with a smaller likelihood of returning. In terms of the success–failure dichotomy, we could therefore safely say that, among these young adults who moved to large cities—and to the extent that the success–failure dichotomy is relevant—return migration is more likely a sign of failure than a sign of success. It should be stressed that this finding may indeed be specific to young adults, also because a sizeable proportion return not only to the home region but also to the parental home. Furthermore, particularly among students, returning to the parental home could be a temporary phase in a period of transitioning from education to work rather than a sign of success or failure.

The findings are in line with theoretical ideas on the importance of family members in social networks and support exchange, as expressed in, for example, the literature on family solidarity. They could indicate that siblings and parents living nearby, in the same city, facilitate integration in the city after migration. Potentially, this implies that those with family in the city have better opportunities to benefit from the options the city offers in terms of education and jobs. Conversely, this might mean that those from one‐child families or single migrants from otherwise sedentary families could have lesser chances to benefit from these options.

The findings could also indicate that return migration is associated with seeking companionship of siblings or parents living in the home region, tapping into the parents' social capital or resorting to family in times of need. Because our data do not give us any clue on actual interaction with the siblings and parents, we have to be careful interpreting the findings in these terms. It cannot be completely ruled out that the associations we found are coincidental and that the locations of parents and siblings stand for something else—for example, attachment to the place of origin, the broader social network, or high school friends. Yet, previous findings from in‐depth interviews with both return migrants and non‐returnees suggest that particularly parents, and siblings, form important considerations in decisions on whether or not to return to a home region (Von Reichert et al., 2013). Furthermore, research using Swedish data on migration motives showed that, among those movers who lived over 50km from a parent or child before the move and within 20km afterwards, over half mentioned family as a motive for the move (Gillespie & Mulder, 2020). We therefore think it is highly unlikely that our findings arose from pure coincidence.

A few findings not related to our hypotheses are noteworthy. Those young adults whose parents no longer lived in the home region were more likely to migrate elsewhere than those whose parent(s) still lived there. It could be that some of these migrated to a region where a parent had moved or where the partner's parents lived. We found that young adults whose parents were highly educated or had a high income were more likely to migrate elsewhere but not more likely to return. Consequently, it seems likely that resourceful parents encourage or facilitate onward migration but not return migration.

We were lucky to have access to register data for the entire population of Sweden. Such data offer opportunities to investigate phenomena that are difficult to investigate using survey data. It is indeed very difficult to think of a survey design in which migration from large cities, and particularly the role of parents and siblings in this migration, could be studied without running into small‐N problems. Even with our register data, we occasionally encountered numbers not greater than several tens—for example, of young adults moving back to a region in which one or more siblings lived but no parent. The availability of parent–child links is very fortunate. Other advantages of register data are efficiency in data collection, absence of respondent burden and non‐response, and a very low number of missing values.

Yet, register data also have their limitations. The reliability depends on what is registered and on how accurately inhabitants of a country report information to the register, for example, how accurately they report changes of address. Although there are strong incentives to report changes of address in Sweden, particularly young adults may fail to do so. Another problem is incomplete information about education and links to adult family members for those born abroad, which is why we had to leave non‐Swedish born out of this study. In the case of Sweden before 2011, information about unmarried cohabitation among those without common children is also lacking. Because cohabitation is very common in Sweden, this also implies we have no reliable information on separation of the young adults. Neither did our dataset include information on housing.

We see many options for further research and name just a few. With the data at hand, it would be possible to investigate return migration from other areas in Sweden than just the large cities. The age range could be extended or shifted towards older ages. This would allow investigating the role of family in “stepping off the escalator” after social mobility (Champion, 2012; Fielding, 1992). It would also open the option to incorporate the support needs of the parents arising from, for example, divorce, widowhood (Smits, 2010; Smits et al., 2010; Thomas & Dommermuth, published online before print) or old age. To shed more light on the association between return migration and moving towards parents, another topic of investigation could be migration towards parents who do *not* live in the home region. It would also be interesting to investigate the role of nearby siblings in economic outcomes such as educational attainment, income, and employment. With other sets of register data, it would be possible to include information about separation, housing, and health. With data in which partners can be identified in a better way (Swedish register data for more recent periods or register data for other countries), it would also be possible to consider family members of the partner (Albrecht, Döring, Holz‐Rau, & Scheiner, 2019). One might even think of using data on neighbours, classmates, or military service cohorts to approximate social networks.

As a matter of course, many other topics related to ours cannot be investigated with register data. An example is how migration is related to actual interactions between family members and other social network members, including contact or support exchange. There is some previous work in this area (e.g., Ermisch & Mulder, 2019; Hünteler & Mulder, published online before print), but small‐N problems are easily encountered. Yet, because register data are only available for a limited number of countries, survey data are the only feasible option to study the role of non‐resident family in migration using quantitative methods. Such data might offer the opportunity to do some work for more familistic countries such as Spain or Italy. Qualitative research is also an option. In this respect, Von Reichert et al.'s (2011, 2013, 2014) research design deserves to be mentioned. She and her colleagues conducted short in‐depth interviews with attendants of high school reunions. This allowed them to discuss the attractions of the home region with stayers, return migrants, and non‐returnees alike and to obtain valuable information about motives for staying, returning, and not returning.

Despite the limitations of our research, we think we have provided convincing evidence of the importance of siblings and parents in young adult migration. The fact that this evidence was found for Sweden—known for not being a familistic society—raises curiosity about the role of non‐resident family in other contexts.

## CONFLICT OF INTEREST

The authors report no conflict of interest.

## Notes

1 Some text parts in this section were copied or adapted from Mulder et al. (accepted for publication).

2 These were the years for which the data had been released by Statistics Sweden and purchased by the institution at which we carried out the research (2013 was also available but could only be used for measuring migration).

3 Although the coverage of internal moves in the Swedish register data is good in general, there is a known minor underestimation of students' moves (Linköpings Kommun, 2014).

^†^
*p* < .10

*
*p* < .05

**
*p* < .01

***
*p* < .001
